# A Customized Vision System for Tracking Humans Wearing Reflective Safety Clothing from Industrial Vehicles and Machinery

**DOI:** 10.3390/s141017952

**Published:** 2014-09-26

**Authors:** Rafael Mosberger, Henrik Andreasson, Achim J. Lilienthal

**Affiliations:** AASS Research Centre, Örebro University, 70182 Örebro, Sweden; E-Mails: henrik.andreasson@oru.se (H.A.); achim.lilienthal@oru.se (A.J.L.)

**Keywords:** infrared vision, human detection, industrial safety, high-visibility clothing

## Abstract

This article presents a novel approach for vision-based detection and tracking of humans wearing high-visibility clothing with retro-reflective markers. Addressing industrial applications where heavy vehicles operate in the vicinity of humans, we deploy a customized stereo camera setup with active illumination that allows for efficient detection of the reflective patterns created by the worker's safety garments. After segmenting reflective objects from the image background, the interest regions are described with local image feature descriptors and classified in order to discriminate safety garments from other reflective objects in the scene. In a final step, the trajectories of the detected humans are estimated in 3D space relative to the camera. We evaluate our tracking system in two industrial real-world work environments on several challenging video sequences. The experimental results indicate accurate tracking performance and good robustness towards partial occlusions, body pose variation, and a wide range of different illumination conditions.

## 1. Introduction

Many of today's industrial work environments are organized as shared work spaces in which vehicles and machinery operate in close proximity to human workforce. The absence of safe separate work zones exposes the workers to a number of risks, namely accidents, injuries and fatalities due to contact collisions with moving vehicles. With the aim of increasing the safety for operators and co-workers and preventing work-related accidents, the industry has shown an increasing interest in on-board safety technology that is able to automatically detect, locate and track workers in a specific hazardous zone around the host vehicle. Situational awareness about the presence and exact location of humans with respect to the machine is crucial for different reasons. On the one hand, it plays an important role in the development of driver assistance technology for human operated machinery. On the other hand, it is even a fundamental prerequisite for safe navigation, path planning and collision avoidance for either semi-autonomous or fully autonomous robots operating in the vicinity of humans.

However, reliably detecting and tracking humans in an industrial environment is complex and challenging. Many industrial work sites are cluttered spaces in which humans are often partially occluded. The environment is often highly dynamic, with the humans to be tracked, the machines and possibly other objects and materials in motion. Industrial machines also operate often in both indoor and outdoor environments and are confronted with a wide range of different weather and illumination conditions. Especially in outdoor applications, vehicles also frequently face rough terrain, entailing further camera motion and therefore presenting additional tracking challenges. Workers to be detected appear at various distances and angles and the non-rigid, articulated nature of the human body leads to a considerable intra-class variation.

While similar requirements in the field of road traffic safety have led to the development of advanced pedestrian protection systems, their adaption from the use in cars to the use in industrial machines has so far attracted much less attention. Approaches to reliable detection of industrial workers have been based on various sensor modalities, including visible-light and infrared imaging, time-of-flight sensors such as radar and lidar as well as radio frequency technologies including Radio Frequency Identification (RFID), Ultra Wideband (UWB) and the Global Positioning Systems (GPS). However, within the context of industrial safety, every technology suffers from certain limitations in terms of either reliability, availability or applicability in operational conditions.

**Figure 1.**
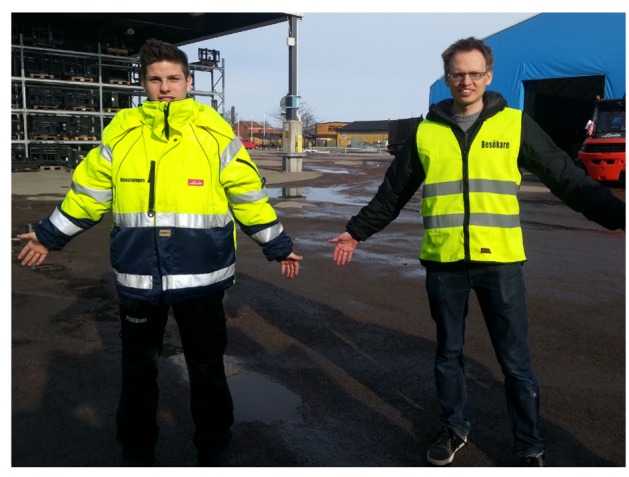
The two types of reflective safety garments used throughout the experiments: ANSI/ISEA safety class 2 vest with reflectors only in the body area (**right**) and safety class 3 jacket with additional reflectors in the shoulder and arm area (**left**).

Our work therefore focuses on the task of real-time on-board human tracking from construction and transportation machinery that operates in industrial environments such as manufacturing areas, construction sites, warehouses, or storage yards. We thereby exploit the fact that high-visibility clothing has become widely accepted throughout industry as an effective way to protect industrial workers that are exposed to heavy vehicles and equipment. The most frequent types of such protective safety garments used in industry are vests and jackets in fluorescent color, with parts of their surface covered with retro-reflective strips (see Figure 1). The reflectors on the safety clothing considerably increase the worker's visibility for an observer located near a light source. Based on this principle, we present a novel vision-based human tracking approach whose core idea consists in tracking industrial workers purely by detecting the retro-reflective markers attached to their safety clothing. Our approach is based on the input of a specifically designed monochrome stereo camera setup with active IR illumination that, at the basis, allows the system to detect, locate, track and classify any dynamic object with highly retro-reflective properties. We focus on the task of human detection by learning the reflective appearance model of safety garments in supervised manner. No assumptions on the human body posture are made, as we specifically aim to detect workers carrying out arbitrary actions. In addition, we don't assume the presence of a flat ground-plane in order to keep our method applicable for any application on rough outdoor terrain.

We first introduced our principal approach of detecting the reflectors of safety vests using a monocular camera setup, equipped with a flash unit [1]. It was shown that by acquiring pairs of images, one using flash and one without, reflective material can be identified by considering the intensity difference between corresponding image regions of the acquired image pair. The method was then extended to illustrate that by using supervised learning of state-of-the-art image feature descriptors extracted from the reflective interest regions, we are able to infer two useful quantities [2]. First, a classifier was trained to assign a class label to the detected reflective patterns, in order to discriminate between reflectors of a reflective garment and other types of reflectors. Second, the metric distance between the camera and a reflector was estimated with an accuracy of less than one meter using supervised regression. The output of the classifier and regressor were then combined to perform real-time tracking of a single person using a particle filter [3]. The method was further extended in order to address the more challenging task of simultaneously tracking multiple humans and the monocular camera was replaced by a stereo setup [4].

This article makes the following novel contributions. (1) We review, summarize and consolidate the research on our industrial human tracking system while offering a more exhaustive discussion of the individual parts of the system; (2) We present an extended evaluation of the system and its constituent parts using challenging real-world data from two different industrial environments; (3) We provide a discussion of the experimental results and highlight the advantages and limitations of our method in comparison with other vision-based human tracking approaches.

The remainder of the article is structured as follows. In Section 2 we review related work in the domain of human detection and tracking in an industrial context. Section 3 then discusses both the physical camera setup as well as the individual processing steps of the algorithm that build our industrial human tracking system. In Section 4 we show experimental results on a set of challenging video sequences acquired in different real-world industrial environments while a discussion of the results is presented in Section 5. Finally, Section 6 summarizes the paper and gives an outlook on future work.

## 2. Related Work

Our work is closely related to the field of pedestrian detection and tracking for road safety. With the aim of protecting pedestrians, today's cars are increasingly equipped with safety technology in the form of pedestrian protection systems (PPSs). The aim of a PPS is to detect humans in a specific area of interest around the host vehicle and mitigate dangerous situations by either providing the driver with alert signals or automatically taking counteractive measures, such as breaking the vehicle. A comprehensive overview of the vast literature about sensors and algorithms used in pedestrian protection systems is out of the scope of this article, and we refer the reader to the recent surveys by [5–9]. Here, we confine the review of PPSs to the most important points related to our work on industrial human detection.

The major part of approaches towards on-board pedestrian detection and tracking for the use in a PPS rely on the input of cameras that operate either in the visible, near infrared (NIR) or thermal infrared (TIR) domain. In addition, approaches that combine vision-based methods with alternative sensor modalities including LIDAR and RADAR have been investigated. For analyzing the different algorithms in use, the architecture of a PPS can be separated into several key modules that appear in different configuration in the majority of the existing systems. The modules that are most related to our work are foreground segmentation, object classification and tracking. Foreground segmentation aims at extracting regions of interest from the raw input data. Vision-based approaches often exploit various cues such as color or intensity, texture, stereo [10], or optical flow [11]. Other algorithms use no explicit segmentation and perform exhaustive scanning over the entire image, such as the popular HOG detector [12]. Popular object classification methods used in PPSs include template-based silhouette-matching [10]), body part models [13], or classification of appearance based feature descriptors such as HOG [12]. Tracking modules operate either in the 2D image space or in 3D camera coordinates and frequently make use of Mean Shift Tracking [14], Extended Kalman Filters [15] or particle filters [16].

In contrast to the field of road traffic, robust human detection from vehicles operating in industrial environments has so far attracted less attention in research. Although the principal objective of providing robust on-board human detection is the same, there exist numerous differences between the two fields that make the direct industrial application of existing PPSs difficult. Most importantly, the operational conditions that industrial vehicles typically face are more challenging. They often operate alternately in bright outdoor and poorly illuminated indoor areas, which leads to a broad range of different illumination conditions, a fact that hampers the adoption of purely visible-light vision based detectors that rely on good image contrast or texture. Especially in outdoor applications, they are also often faced with rough terrain. In consequence, the flat floor assumption is often not valid and detections cannot be restricted to certain image regions by introducing ground plane constraints. When working with thermal vision sensors, the presence of various heat sources in an industrial work site makes the extraction of foreground regions more difficult than in road traffic. Further challenges also include the large variety in human body pose that is encountered in an industrial work place. Instead of observing mainly upright pedestrians as is the case in road traffic, industrial workers can appear in a number of different body positions depending on the activity that is performed. Furthermore, the typical motion scenarios of industrial vehicles comprise more acceleration and deceleration, sharper turns and reversing, resulting in dangerous front, rear and lateral zones. Consequently, the desired sensor coverage for human detection is bigger than the relatively narrow cone observed in front of a regular car.

Considering the above challenges, various attempts have been made to addressed the problem of human detection and tracking in an industrial context. Color vision in combination with various tracking algorithms was successfully used to track and monitor construction workers from both static and dynamic cameras [17]. A pedestrian detection system specifically designed for underground mining vehicles [18] proposes the use of thermal images for detection and a 3D range data for depth estimation. Thermal vision is used in this case because of its insensitivity to illumination changes and to the frequent obscuring dust in underground mining. Another vision based human detector using a stereo camera was designed for heavy industrial machinery [19]. The authors claim that traditional single detector based approaches are insufficient for the operation in challenging work environments. To overcome the problem, they provide a modular framework that allows them to combine responses from different human detectors in order to increase the system robustness. Radio-frequency identification (RFID) is another popular method for robust proximity warning systems and has been employed to detect humans in the neighborhood of heavy construction machinery [20]. While the approach proves to be effective, workers have to be equipped with costly sensing infrastructure. The specific approach of using the properties of high-visibility clothing in order to facilitate the detection of industrial workers has also been previously investigated [21]. Their algorithm identifies the fluorescent color of safety vests by processing local color histograms extracted from the regions of interest. Even though the reflectivity of the vests is not exploited, the authors show that the distinctive color of safety clothing contributes to a more effective detection.

## 3. System Description

**Figure 2.**
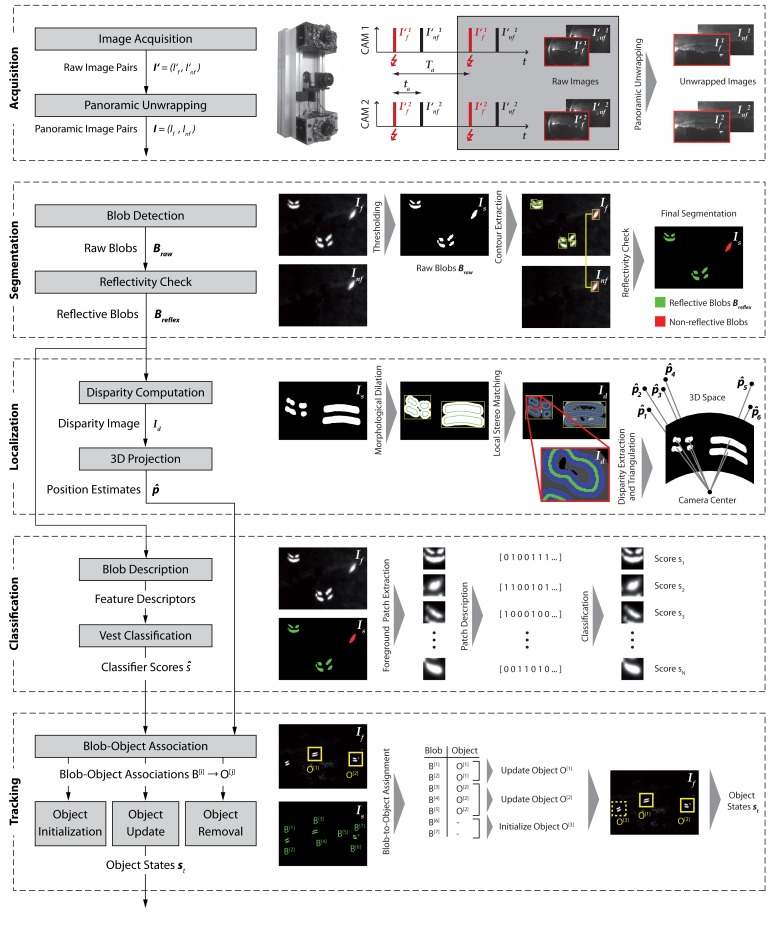
Schematic overview of the different stages of our human tracking system.

**Figure 3.**
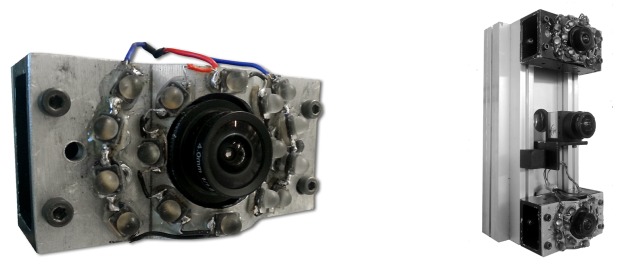
Sensor setup used throughout our experiments: Near-infrared sensitive camera equipped with wide-angle lens, bandbass filter, and 16 high-power IR LEDs (**left**) and stereo camera unit formed by two identical camera units (**right**). The additional color camera in the center of the stereo rig is not used by the tracking algorithm but serves purely for visualization purposes.

**Figure 4.**
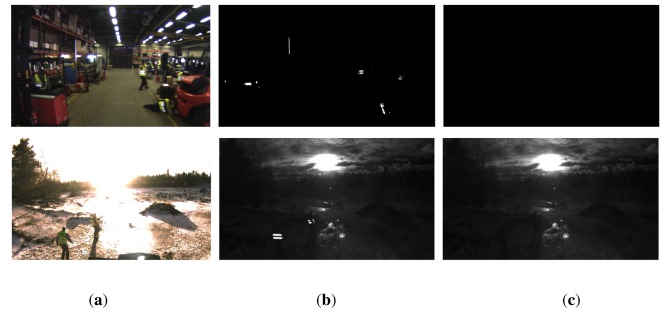
Examples of unwrapped input images as captured by (**a**) a color camera; (**b**) the NIR camera using flash; and (**c**) the NIR camera without using flash. The top row shows images taken in an indoor environment and in the absence of any secondary NIR light source, resulting in a completely dark image background. The bottom row illustrates images captured outdoors under strong backlight conditions, leading to much higher background illumination. Our algorithm exploits the fact that reflective objects appear significantly brighter in the image taken with flash while background illumination is constant over both images.

Figure 2 gives a schematic overview of our proposed vision-based human tracking system. Our core idea is to track industrial workers by detecting the retro-reflective stripes attached to the high-visibility clothing widely used throughout industry. For this purpose, we use a customized monochrome stereo camera setup (*cf.* Figure 3) that is designed to acquire images in which reflectors appear as distinctive high-intensity regions in front of the non-reflective scene background (*cf.* Figure 4b). While a continuous video stream is acquired by the stereo camera, an infrared flash is used alternately for every second image capture. Two consecutively acquired images of each camera are grouped together to form flash/non-flash image pairs and the two obtained pairs then build a group of four images that are processed together by the algorithm. Before entering the processing chain, the four images are unwrapped in order to obtain undistorted images that cover the meaningful, often panoramic field of view around the host vehicle.

The set of reflective foreground regions is then further processed to estimate two important quantities. In a *localization* module, the 3D position relative to the camera is estimated individually for every reflective foreground blob. To do so, we make use of the stereo input and compute a dense disparity map in a local neighborhood around each foreground blob. After extracting a single aggregated disparity value per blob, we infer the metric distance to a reflector as well as the 3D position using triangulation.

In an initial *segmentation* stage, we detect reflective material by extracting image regions showing a clear intensity difference between the two images of a flash/non-flash pair. We thereby exploit the fact that reflectors appear significantly brighter in an image captured with flash, following the strong back reflection. Using this effect, segmentation is performed in two steps. First, we extract a set of high-intensity blobs from the image captured with flash by performing local adaptive thresholding. We then submit each of the blobs to a reflectivity check that rejects candidates for which the intensity difference between the two images of a pair is low. However, to calculate the intensity difference, we first need to relate corresponding image regions between the flash and non-flash image. This is necessary because a time delay exists between the acquisition of the two images, and the camera or the observed objects might be in motion.

In a parallel *classification* module, we further assign a class membership to every blob. When operating in a typical industrial environment, it is likely that various reflective objects other than the worker's safety vests are also captured by the camera. Classification of the observed reflective patterns is therefore necessary in order to discriminate reflectors on a safety garment from arbitrary other reflective objects. To obtain a robust blob description, local image feature descriptors are first extracted from the neighborhood of each reflective foreground blob. A classification score is then obtained using a classifier that was previously trained in supervised manner.

In the final *tracking* stage, we then attempt to assign the detected reflective blobs to a set of tracked objects. Every tracked object maintains a filtered estimate of each object's 3D position and speed relative to the camera using a particle filter that is fed with the individual blob-level 3D position estimates. We thereby specifically track all reflective objects regardless of their nature. A final estimate whether or not a human wearing a reflective safety garment is observed is inferred by aggregating the individual classification scores of all blobs assigned to a given object.

### 3.1. Hardware and Camera Model

Our camera setup is specifically designed for the task of detecting retro-reflective material. With the combination of a monochrome camera, optical filter, and active illumination we pursue the objective of separating the appearance of reflective and non-reflective material already to a great extent at the moment when the images are acquired. This allows us to significantly reduce the complexity of the subsequent segmentation techniques applied to extract the image regions corresponding to reflective objects.

With the configuration of our camera setup we pursue two primary goals concerning the characteristics of the images that are acquired. First, we want retro-reflective material to appear as distinctive, saturated high-intensity image regions by equipping the camera with a flash unit. The intensity of the flash and camera exposure time are chosen such that retro-reflectors appear fully saturated in the image. Second, we aim to reduce the illumination of the non-reflective image background to the extent possible. This is achieved by including a narrow optical bandpass filter that removes all wavelengths other than the ones corresponding to the emitted flash. We thereby choose to operate the camera setup in the near-infrared (NIR) range in order to make the active illumination unobtrusive.

**Figure 5.**
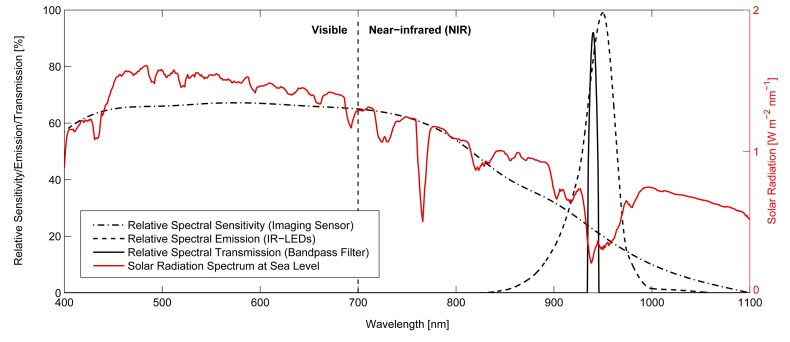
The figure shows the relative spectral characteristics of the camera, the bandpass filter and the flash diodes. The red curve represents the solar irradiation spectrum at sea level. The operation wavelength of 940 nm is chosen to exploit the negative peak in the sun spectrum. Especially in outdoor applications, this allows us to considerably reduce the undesired background illumination.

The particular setup used throughout our experiments (*cf.* Figure 3 left) consists of an 1-megapixel off-the-shelf monochrome CMOS sensor (IDS Imaging GigE UI-5240CP-NIR) with high NIR sensitivity. A wide-angle lens with a field-of-view of 120° is chosen to cover the desired region of interest around the host vehicle. 16 highpower (1.8 W) infrared LEDs with a centroid wavelength of 940 nm and a fullwidth at half maximum (FWHM) of 35 nm are placed close to the lens. The spatial arrangement and orientation of the LEDs provide a relatively uniform illumination over the entire FOV of the camera. Finally, a bandpass filter with a centroid wavelength of 940nm and a FWHM of 10nm is mounted between the lens and the sensor. The spectral sensitivity, emission and transmission curves for the sensor, the LEDs and the filter are depicted in Figure 5. The choice of operating with wavelengths around 940 nm is motivated by a strong negative peak in the sun spectrum at this point, caused by atmospheric gas absorption. This allows us to reduce the disturbing effect of background illumination observed when the camera is exposed to the sun.

Using two identical camera units, we then build a stereo setup with a base line of 200 mm (*cf.* Figure 3 right). The stereo rig is further equipped with a color camera that is used purely for visualization purposes. The vertical arrangement of the two cameras accounts for the panoramic unwrapping process described in the next section and preserves the existence of straight epipolar lines.

In order to provide full flexibility to work with different lens types, including wide-angle, fish-eye and omni-directional, we propose to adopt an omnidirectional camera model [22]. The adopted model defines an image projection function ***g*,** describing the relation between a 2D image coordinate pair ***u′*** = [*u′*, *υ′*]^⊤^, the metric coordinates ***u″*** = [*u″*, *υ″*]^⊤^ on the sensor plane, and a unit length 3D vector emanating from the camera's optical center *O* to the according scene point in 3D space,

(1) $$\text{g}(A{\text{u}}^{\prime }+\text{t})=\text{g}({\text{u}}^{\prime \prime })={\left(u^{\prime \prime },\upsilon^{\prime \prime },f(u^{\prime \prime },\upsilon^{\prime \prime })\right)}^{⊤}$$

with *f* a polynomial function, rotationally symmetric with respect to the sensor axis. The affine transformation *A****u′*** + ***t*** accounts for the digitizing process as well as small axes misalignments.

### 3.2. Image Acquisition and Panoramic Unwrapping

A synchronized image stream is acquired from the stereo camera where images are captured alternately with and without using flash. Two successively acquired images are grouped together and form a flash/non-flash input image pair. The time increment *t_a_* between the capture of the two images of a pair is kept as short as possible in order to minimize the difference between the two images due to both changes in viewpoint under camera motion and changes in the observed scene. The pairs of raw flash/non-flash images of both cameras, denoted $I^{\prime 1}=\left(I_{f}^{\prime 1},I_{nf}^{\prime 1}\right)$ and $I^{\prime 2}=\left(I_{f}^{\prime 2},I_{nf}^{\prime 2}\right)$ for the first and second camera of the stereo unit respectively, represent the sole input to the detection and tracking algorithm.

It is important to note that, in the absence of any secondary NIR light source, it would be sufficient to acquire single images with flash instead of flash/non-flash image pairs. As illustrated in Figure 4 (top row), the image taken under active illumination shows bright reflectors in front of an entirely dark background. In this ideal case, image segmentation would be achieved by simple thresholding. However, this approach fails if the scene background is subject to external NIR illumination as it happens for example under exposure to strong sunlight. Figure 4 (bottom row) illustrates that, in this case, reflective material no longer represents the only bright image regions. Therefore, the image taken without active illumination serves as a reference image for the background illumination.

**Figure 6.**
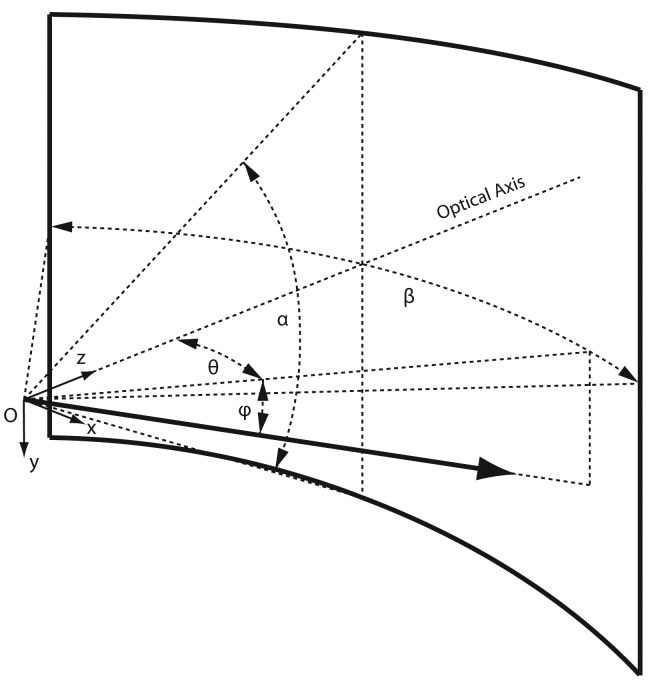
Parametrization of the virtual cylinder used to define the panoramic field of view for unwrapping of the raw images. The reference (*x*, *y*, *z*) indicates the orientation of the coordinate system attached to the camera.

After acquisition, the raw images $I_{f}^{\prime }$ and $I_{nf}^{\prime }$ are unwrapped to create a pair of undistorted panoramic images ***I*** = (*I_f_*, *I_nf_*), containing the desired area of interest. Figure 6 illustrates the virtual cylinder with unit radius defining the panoramic field of view. The relationship between a pair of panoramic image coordinates ***u*** = [*u, υ*]^⊤^ and a unit length vector pointing to the according point in 3D space is defined by the image projection function ***h*,**

(2) $$\text{h}(\text{u})=\left(\begin{array}{c} \cos\phi \sin\theta \\ -\sin\phi \\ \cos\phi \cos\theta \end{array}\right)$$

with the azimuth angle *θ* and the altitude angle *ϕ* defined by

(3) $$\begin{array}{ccc} \theta =\beta \left(\frac{u}{W-1}-\frac{1}{2}\right) & \text{and} & \phi =\tan^{-1}\;\left[\tan\frac{\alpha }{2}\left(1-\frac{2\upsilon }{H-1}\right)\right] \end{array}$$

while the panorama image width *W* and height *H* are related through $H=2\tan\left(\frac{\alpha }{2}\right)\beta^{-1}W.$

### 3.3. Blob Detection and Reflectivity Check

In a first stage, we perform a segmentation of the input images with the objective of identifying retro-reflectors in the image and extract the contours around them. Our approach is based on three major observations made from inspecting typical input images as depicted in Figure 4.

1. The retro-reflective stripes we aim to detect appear significantly brighter in image *I_f_* when compared to *I_nf_*, due to the strong back reflection of the emitted flash.
2. Other bright objects in the images, such as the sun, or surfaces that are illuminated by the sun or another secondary IR light source, have similar intensity values across the images *I_f_* and *I_nf_*.
3. As a time difference *t_a_* exists between the acquisition of *I_f_* and *I_nf_*, the exact location of an object is not necessarily the same in both images. This applies if either the observed object in the scene or the camera itself are in motion.

The first two observations indicate that to detect reflective material in an input image pair, we need to identify image regions with considerably higher intensity values in *I_f_* than in *I_nf_*. However, due to the third point, a direct subtraction of image *I_nf_* from *I_f_* is not an adequate way to detect such regions. Instead, we proceed by first applying local adaptive thresholding to image *I_f_*, leading to a binary image *I_s_* containing a set of high-intensity foreground regions. The adaptive threshold is computed as the mean intensity value in a square local neighborhood of size *w_th_* × *w_th_* subtracted by an offset *c_th_* > 0. A suitable choice of the parameters *c_th_* and *w_th_* assures that the segmented foreground regions always include the reflectors to be detected. Using contour following according to [23] applied to image *I_s_*, we then extract a set of raw blobs 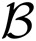
_raw_ in which each blob *b* is characterized by its respective contour Λ and the center ***u****_c_* = [*u_c_*, *υ_c_*] of the contour's bounding rectangle:

(4) $${\text{B}}_{\text{raw}}=\left\{b^{\left[i\right]}=\langle \Lambda^{\left[i\right]},{\text{u}}_{c}^{\left[i\right]}\rangle |i=1,\ldots ,N_{\text{raw}}\right\}$$

The detected blobs in set 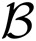
_raw_ are then submit to a reflectivity check. The test verifies whether the intensity values in the surrounding area of a blob are similar in the images *I_f_* and *I_nf_*, or if they are instead distinctly higher in image ***I****_f_*. The first case is an indication that the blob corresponds to an object that appears bright due to background illumination by a secondary IR light source. Blobs that match this criterion are rejected. In contrast, the second case indicates that the corresponding object has highly reflective properties and therefore the blob is retained.

The test is performed by first computing the bounding rectangle around the contours of all raw blobs *b* ∈ 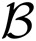
_raw_ extracted from image *I_f_*. We then apply the pyramidal implementation of the optical flow based iterative Lucas-Kanade (LK) feature tracking method [24] to determine the position in image *I_nf_* that corresponds to the region delimited by the bounding box in the image *I_f_*. Especially under fast rotational camera movements, this positional offset can amount to several pixels, due to the time delay *t_a_* in the acquisition of *I_f_* and *I_nf_*.

The LK tracker is based on the assumption that the brightness of an object is constant over two consecutive frames. In the case of non-reflective objects where the appearance in *I_f_* and *I_nf_* is very similar, this assumption holds true. The LK tracker typically finds the corresponding location in image *I_nf_* after a small number of iterations. In this case, we compute the local average intensity difference Δ*I* = *I_f_* − *I_nf_* within the bounding rectangle, taking into account the rectangle's respective location in *I_f_* and *I_nf_*. A low intensity difference Δ*I* is then a confirmation that the appearance of the blob in *I_f_* and *I_nf_* is similar, and a strong indication that the concerned blob represents a non-reflective object. In the contrasting case of a reflector, the assumption of constant brightness over *I_f_* and *I_nf_* is obviously violated, because intensity values are clearly higher in *I_f_*. Here, the tracker is most often unable to find any suitable match in the image *I_nf_*.

**Figure 7.**
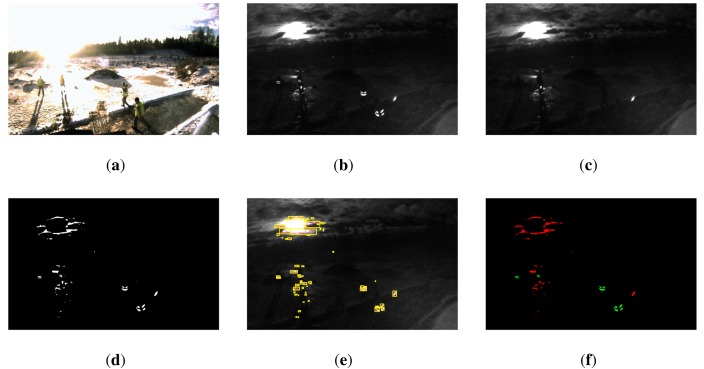
Illustration of the segmentation process for an input image pair acquired in the challenging case of strong backlight conditions. (**a**) Observed scene as captured by a color camera; (**b**) NIR image *I_f_* captured with flash; (**c**) NIR image *I_nf_* captured without flash. (**d**) Image *I_s_* obtained after applying adaptive thresholding to image *I_f_*; (**e**) Bounding boxes (yellow) around the segmented high-intensity regions. Optical flow based Lucas-Kanade tracking is applied to determine the position of the indicated regions in image *I_nf_*. A reflectivity check then assesses whether a clear intensity difference exists between the two images; (**f**) Fully segmented image, with reflective parts marked in green and non-reflective parts in red.

Based on the above observations, we use the output of the LK tracker and the intensity difference check to split the initial set of blobs 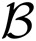
_raw_ into two subsets 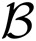
_reflex_ and 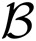
_non-reflex_ according to

(5) $${\text{B}}_{\text{non}-\text{reflex}}=\left\{B\in {\text{B}}_{\text{raw}}|B\;\text{tracked}\wedge \Delta I<\lambda_{\Delta }\right\}$$

(6) $${\text{B}}_{\text{reflex}}={\text{B}}_{\text{raw}}\backslash {\text{B}}_{\text{non}-\text{reflex}}$$

where 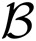
_reflex_ is assumed to contain only features originating from reflective material. Here, it is worth noting that in contrast to the standard application of a feature tracker, we are not only interested in features that can be successfully tracked from one image to the other. Instead, we specifically identify features for which LK tracking fails and assume that the reason for the failure is the different appearance of the feature over both images. Figure 7 illustrates the described image segmentation procedure in the case of strong backlight conditions.

### 3.4. Local Disparity Computation and 3D Projection

The goal at this stage is to estimate the 3D position of all detected reflective objects represented by the set of foreground blobs 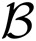
_reflex_. We therefore make use of the stereo image pair $\left(I_{f}^{1},I_{f}^{2}\right)$ and perform dense stereo matching before extracting one single aggregated disparity value per blob. We thereby limit disparity computation to a close neighborhood around the image area covered by the blobs *B* ∈ 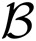
_reflex_. This considerably reduces the computational effort compared to dense stereo matching over the entire image.

**Figure 8.**
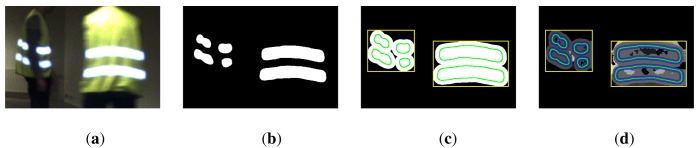
Disparity computation for a set of foreground blobs: (**a**) Observed scene as captured by a color camera; (**b**) Segmented foreground image *I_s_*; (**c**) Connected foreground clusters obtained after morphological dilation. The bounding rectangles (yellow) indicate the area in which dense stereo matching is performed. The original contours of the blobs are drawn in green; (**d**) The final disparity image. The blue zone around the blob contour indicates the area in which disparity values are aggregated to obtain an overall disparity estimate per blob. Black areas within the bounding rectangles indicate regions where the matching fails, due to weak texture. Deviating gray levels inside the blob region indicate erroneously estimated disparities due to mismatched pixels.

The procedure is illustrated in Figure 8 and departs from the segmented foreground image *I_s_* from which we have removed all non-reflective blobs. In a first step we apply morphological dilation in order to merge neighboring blobs and create clusters of foreground blobs. From the resulting binary image we extract the bounding rectangles around the extended and possibly merged foreground components. Dense stereo correspondences are then computed within the areas covered by the rectangles using the semi-global block matching algorithm [25]. Due to the lack of sufficient texture in the image pair $\left(I_{f}^{1},I_{f}^{2}\right)$, disparities are often not unambiguously computed for the entire area within the bounding box.

In addition, mismatched pixels lead to erroneous disparity values, an effect that applies especially for the saturated white regions within a blob. However, due to the clear-cut edge between the bright surface within a blob and the dark surrounding, stereo matching is consistently and accurately achieved in the regions surrounding the contour Λ of a blob. We therefore consider all disparity values computed at pixels with a distance smaller than *s* from the blob's contour Λ and use the median value as an aggregated disparity estimate for a blob.

We then use the assigned median disparity to triangulate the metric distance *d* to the object represented by a foreground blob. Using the projection function ***h***(***u***) of our virtual cylindrical camera model applied to create the panoramic images (Equations (2) and (3)), we further obtain a 3D position estimate ***p̂***:

(7) $$\hat{\text{p}}=d\times \text{h}(\text{u})=\frac{b\times f}{D\cos(\phi )}\times \text{h}({\text{u}}_{c})$$

where *f* = 1 is the focal length of our virtual cylinder, *b* the baseline of the stereo camera, and *D* the disparity expressed in metric units on the virtual cylindric image plane.

### 3.5. Blob Description and Classification

The output of the previous processing steps is a set of blobs 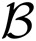
_reflex_ that we assume to originate from reflective objects in the observed scene. While our goal is to detect the reflectors of a worker's safety garment, other reflective items are also frequently encountered in an industrial environment. Typical examples are cat's eye reflectors on vehicles, metallic surfaces, windows, mirrors, and various types of reflective signs attached to walls, floors, or materials. To specifically detect workers wearing a reflective safety vest and prevent false alarms in case of any other reflective object, it is important to discriminate between the two cases, and we therefore introduce a binary classification step. Classification is performed individually for every reflective foreground blob *B* ∈ 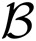
_reflex_. Instead of determining a simple class membership, we opt to produce a continuous class score *ĉ*. Classification is then achieved by thresholding according to the score.

To perform the classification, we first extract an image feature descriptor from each blob's local neighborhood. On one hand, the extraction of a descriptor serves to considerably reduce the dimensionality of the subsequent classification problem. On the other hand, we attempt to obtain a blob description that is robust towards image noise and illumination changes. As we have observed earlier [2,3], popular image feature descriptors based on local intensity differences such as SURF [26], BRIEF [27], BRISK [28] or FREAK [29] comply well with our specific requirements and provide good discriminative power to predict the class to which a reflector belongs. The blob descriptors are extracted from an area around the blob's center position ***u****_c_*, while the size of the area is chosen to cover the visual content inside a blob's bounding rectangle.

The classifier models are obtained using supervised learning, where the training data contains both reflectors of different reflective safety clothing as well as a large set of other reflectors captured in various industrial environments. Both a Support Vector Machine [30] and a Random Forest [31] classifier have been employed to perform the classification. The computation of the classification score *ĉ* is dependent on the type of classifier in use. In the case of a Random Forest, the score is calculated as the proportion of decision trees in the forest that classified as sample belonging to a reflective safety garment. In the case of a Support Vector Machine, the signed distance between the sample to be classified and the separating hyperplane is used as the score.

### 3.6. Data Association and Tracking

We again point out that our tracking approach solely relies on observing the reflectors of reflective safety clothing. Most standardized safety garments available on the market feature a pattern of multiple reflectors (*cf.* Figure 1). In consequence, multiple reflective foreground blobs are typically detected for a single observed person while performing image segmentation as described in Section 3.3. In addition, a single reflector might be observed as multiple fragments under the presence of an occluding element. The latter effect is illustrated in Figure 8a,b, where the left person's arm occludes the reflectors on the safety vest and let's them appear as four distinct blobs in the segmented foreground image. Based on this observation, we choose to first regroup neighboring blobs by assigning them to a set of tracked objects and then maintain a filtered estimate of each object's state. Here, we specifically use the term object to emphasize that in a first stage we choose to track all reflective objects regardless of their nature. Only in a second stage, we infer an estimate whether an object represents a person by aggregating the classifier scores *ĉ* provided individually with every assigned blob.

Therefore, let us assume that at a time instant *t* we are given a set of foreground blobs 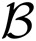
_reflex,_*_t_* representing all detected reflective items in the scene. For each blob a 3D position estimate ***p̂****_t_* and a classification score *ĉ* has been computed:

(8) $${\text{B}}_{\text{reflex},\text{t}}=\left\{{\text{B}}_{t}^{\left[i\right]}=\langle \Lambda_{t}^{\left[i\right]},{\text{u}}_{c}^{\left[i\right]},{\hat{c}}_{t}^{\left[i\right]},{\hat{\text{p}}}_{t}^{\left[i\right]}\rangle |i=1,\ldots ,N_{t}\right\}$$

In a first step we attempt to assign the blobs in 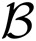
_reflex,_*_t_* to a set 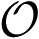
*_t_* of objects being tracked at time instant *t*, using both 2D overlapping and 3D metric distance criteria. Then, based on the assignments, we update the object states and initialize new objects for blobs that failed to be assigned to an existing tracking target. The objects in 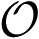
_*t*_ are characterized by their 3D position and velocity state ***s****_t_*, a tracking consistency measure *μ_t_* and an aggregated classification score *c_t_* that the tracked object corresponds to a person:

(9) $${\text{O}}_{t}=\left\{O_{t}^{\left[j\right]}=\langle s_{t}^{\left[j\right]},c_{t}^{\left[j\right]},\mu_{t}^{\left[j\right]}\rangle |j=1,\ldots ,M_{t}\right\}$$

The state vector ***s****_t_*, consisting of the objects 3D position ***p****_t_* and velocity ***ṗ****_t_*,

(10) $${\text{s}}_{t}={\left[{\text{p}}_{t}\;{\dot{\text{p}}}_{t}\right]}^{⊤}={\left[x_{t},y_{t},z_{t},{\dot{x}}_{t},{\dot{y}}_{t},{\dot{z}}_{t}\right]}^{⊤}$$

is recursively estimated by a particle filter. The consistency measure *μ_t_* is used to assess to which extent an object is continuously detected over a series of frames. It is initialized with *μ_t_* = 1 when an object is created. We then increment it by 1 in every frame where at least one blob is assigned to the object, and decrement it by 1 in the opposite case. Objects are first initialized in a passive state as long as their consistency measure *μ_t_* is lower than an activation threshold *λ_μ_*. The class membership score *c_t_*, which reflects the chance that an observed object represents a person, is calculated as the average of the corresponding estimates *ĉ* of all individual blobs assigned to the object up to time *t*. At a given time instant *t*, an object is considered as a reflective safety garment if the score *c_t_* is higher than a threshold *λ_vest_*.

Every object is further represented by a 2D bounding box in the image plane which is used to define a blob-object assignment criterion based on the overlap. The bounding box center thereby represents the back-projection of the object position ***p****_t_* on the image plane. The size of the bounding box depends on the distance of the tracked object and is chosen as the typical size of the human torso.

Our implementation of the particle filter uses the standard sequential importance resampling algorithm [32]. The algorithm sequentially incorporates the obtained per-blob position estimates ***p̂****_t_* by first predicting the object state transition from ***s****_t_*_−1_ to ***s****_t_* using the motion model before assigning each particle an importance factor according to the measurement model. Particle resampling is then performed according to the importance factors using a low variance resampler as proposed by [33]. An initial set of particle is generated by uniformly distributing the particles in the state space.

#### 3.6.1. Data Association

As stated above, it is most likely that multiple reflective blobs are detected if a single person with a reflective safety garment is observed. On the other hand, a reflector cannot represent multiple objects at the same time. In consequence, the first tracking step consists of a many-to-one mapping that assigns blobs to currently tracked objects. For every potential assignment of a blob *B*^[^*^i^*^]^ to an object *O*^[^*^j^*^]^ we define a cost function *d*(*B*, *O*) that takes a low value if it is likely that the *i*-th blob represents a reflector of the *j*-th object and a high value in the opposite case.

The cost function is computed based on two criteria. First, the overlap of the blob area with the bounding box of an object in the 2D image plane should be high. This is expressed by the overlap cost function *d_∩_*, defined as

(11) $$d_{\cap }(B,O)=1-\frac{A(B)\cap A(O)}{A(B)}$$

where *A*(*B*) denotes the area delimited by the contour Λ of blob *B*, and *A*(*O*) the area covered by the 2D bounding box of object *O*. At the same time, the absolute difference of the blob's 3D position estimate ***p̂****_t_* and the object's current 3D position ***p****_t_* should be small. This is expressed by the distance cost function *d_δ_*,

(12) $$d_{\delta }(B,O)=1-\exp(-\alpha \times \left\|{\hat{\text{p}}}_{t}-{\text{p}}_{t}\right\|)$$

with *α* a soft threshold that is heuristically chosen. Finally, we define the overall cost function for assigning blob *B* to object *O* as the weighted sum of *d_∩_* and *d_δ_* where the weights *w_∩_* and *w_δ_* allow to give a preference to one or the other criterion:

(13) $$d(B,O)=w_{\cap }\times d_{\cap }(B,O)+w_{\delta }\times d_{\delta }(B,O)$$

a given blob *B*^[^*^i^*^]^ is then assigned to the object *O*^[^*^j^*^]^ for which the lowest assignment cost was computed, under the restriction that this cost needs to be lower than a defined assignment threshold *λ_d_*. If the lowest computed assignment cost is above *λ_d_*, a blob is considered to belong to an object which is not yet being tracked and no assignment is made.

#### 3.6.2. Object Update

Based on the blob-to-object assignments, we proceed to update the particle filter states ***s****_t_* of all tracked objects in *O* ∈ 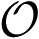
*_t_*. This is achieved by providing each object's particle filter with the assigned blob detections and applying the motion and measurement model, before resampling the particles.

The motion model first predicts the object state transition from ***s****_t_*_−1_ to ***s****_t_* using a description of the system dynamics. In our case, a change of the state ***s****_t_* of an object can be caused either by camera motion or by motion of the observed object itself. We employ a simple constant velocity model of the form

(14) $${\text{s}}_{t+1}=[\begin{array}{cc} {\text{I}}_{3\times 3} & T_{a}\cdot {\text{I}}_{3\times 3}\\ 0_{3\times 3} & {\text{I}}_{3\times 3} \end{array}]\;{\text{s}}_{t}+{\text{w}}_{t}$$

with *T_a_* the inverse of the image pair acquisition rate and ***w****_t_* an independent white noise sequence of the form 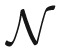 (0, *σ*) referred to as the process noise.

**Figure 9.**
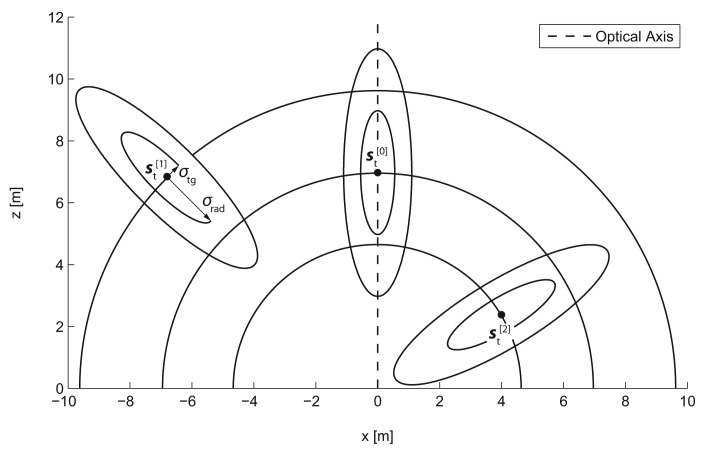
2D projection of the characteristic 3D measurement probability *p*(***p̂****_t_*|***s****_t_*) for three different example states $s_{t}^{\left[0\right]}$, $s_{t}^{\left[1\right]}$ and $s_{t}^{\left[2\right]}$. The camera system is located at the origin. The measurement probability is modeled by a multivariate normal distribution with specific standard deviations *σ_rad_* and *σ_tg_* for the radial and tangential direction. The iso-lines show one and two standard-deviations.

The measurement model then relates the per-blob position estimates ***p̂****_t_* to the object state vector ***s****_t_* by the measurement probability *p*(***p̂****_t_*|***s****_t_*), describing the likelihood of making the observation ***p̂****_t_* assuming that the state of the system is ***s****_t_*. Figure 9 depicts the characteristic shape of the measurement probability *p(****p̂****_t_*|***s****_t_*) in the x/z-plane for three different example states. Due to the processing scheme employed to obtain a position estimate ***p̂****_t_*, the measurement uncertainty is different in the radial and tangential direction and represented respectively by the standard deviations *σ_rad_* and *σ_tg_*. Uncertainty in radial direction mainly originates from the distance estimation error committed by establishing erroneous stereo correspondences. In contrast, the variance in the detection of the tangential position arises from the fact that a detected reflector does not necessarily represent the center position of the observed human. The likelihood of making a single observation ***p̂****_t_*, under the assumption of state ***s****_t_*, is then given by the multivariate Gaussian.

(15) $$p({\hat{\text{p}}}_{t}\text{|}{\text{s}}_{t})=\frac{1}{{\left(2\pi \right)}^{\frac{3}{2}}\text{|}\sum {\text{|}}^{\frac{1}{2}}}\exp\left(-\frac{1}{2}{\left({\hat{\text{p}}}_{t}-{\text{p}}_{t}\right)}^{⊤}\sum^{-1}({\hat{\text{p}}}_{t}-{\text{p}}_{t})\right)$$

where the covariance matrix Σ is obtained using

(16) $$\sum =R_{y}{\left(\theta \right)}^{⊤}R_{x}{\left(\phi \right)}^{⊤}\sum_{0}R_{x}(\phi )R_{y}(\theta )$$

with *θ* and *ϕ* the azimuth and altitude angles of position ***p****_t_*, *R_x_* and *R_y_* the rotation matrices around the x-and y axes respectively and Σ_0_ the covariance matrix corresponding to states ***s****_t_* situated on the camera's optical axis (*cf.* state $s_{t}^{\left[0\right]}$ in Figure 9), given by:

(17) $$\sum_{0}=\left[\begin{array}{ccc} \sigma_{\text{tg}}^{2} & 0 & 0\\ 0 & \sigma_{\text{tg}}^{2} & 0\\ 0 & 0 & \sigma_{\text{rad}}^{2} \end{array}\right]$$

finally, the complete measurement probability defines the likelihood to make the full set of observations $\left\{{\hat{p}}_{t}^{\left[1\right]},...,{\hat{p}}_{t}^{\left[N\right]}\right\}$ corresponding to the 3D position estimates of all blobs assigned to the current object. Under the assumption that the noise in the individual measurements ${\hat{p}}_{t}^{\left[i\right]}$ is independent, it is obtained by the product of the individual measurement likelihoods *p*(***p̂****_t_*|***s****_t_*):

(18) $$p({\hat{\text{p}}}_{t}^{\left[1\right]},\ldots ,{\hat{\text{p}}}_{t}^{\left[N\right]}\text{|}{\text{s}}_{t})\prod_{i=1}^{N}p({\text{p}}_{t}^{\left[i\right]}|{\text{s}}_{t})$$

The weights of all particles in the particle filter are updated according to the above measurement likelihood and resampling is performed. Given the new set of particles, we update the state *s_t_* of every object by computing the weighted mean of all particles. To conclude the object update procedure, we also recompute the other entities hold by an object, namely the classification score *c_t_* and the consistency measure *μ_t_*, both according to the rules described above. We also recompute the 2D bounding box of each object by centering it around the back-projection of the new filtered 3D position estimate *p_t_* on the image plane.

#### 3.6.3. Object Initialization and Removal

Blobs in the set 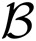
_reflex,_*_t_* that failed to be associated to any tracked object are considered as candidates for the initialization of new objects. To do so, we extract connected regions of similar disparity from the previously computed disparity image and initialize a new *object* for every group of blobs that falls in the same cluster. Finally, existing objects are removed either if their 2D bounding box falls out of the image border or if their consistency measure *c_t_* reaches zero.

## 4. Experimental Results

**Figure 10.**
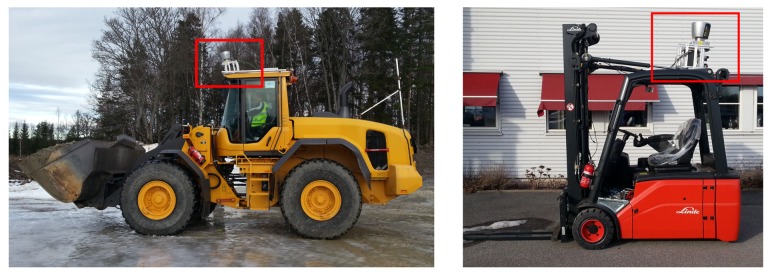
Industrial vehicles on which the camera setup was mounted for the experiments: Wheel loader (**left**) and forklift truck (**right**). The location of the sensor unit with NIR stereo camera and 3D laser scanner (for ground truth data acquisition) is indicated in red.

Our human tracking system was evaluated by performing a set of experiments in two different industrial real-world work environments. The first test environment is an outdoor gravel loading pit where the camera system was mounted on the roof of a wheel loader (*cf.* Figure 10, left). Typical loading and unloading scenarios were simulated, including sharp turns and alternate forward and reverse driving up to 30 km/h. Apart from the host vehicle, a second wheel loader and a hauler were present and a total of four persons were either walking in the area surrounding the machines or operating them. Two test sequences from this environment are evaluated. Sequence 1 was recorded in cloudy weather conditions and contains 3 min of video input. Sequence 2 contains 2 min of video and was recorded in the evening when the sun was low and shining directly into the camera, leading to much higher background illumination. Apart from the reflectors on the safety clothing, the only other reflective objects in the environment were the cat's eye reflectors on the vehicles.

The second test environment is an indoor manufacturing and maintenance site for industrial vehicles. Numerous workers are present in the cluttered area, carrying out tasks in various body postures while often being partly occluded by the various objects in the scene. The sensor unit was mounted on the roof of a forklift (*cf.* Figure 10, right) that was operated at speeds up to 20 km/h. Again, two test sequence are evaluated, both corresponding to two minutes of video input. Sequence 3 represents a scenario in which a considerable amount of reflective objects other than the safety clothing appear. The risk for the system to output false alarms is therefore considerably higher. Seq. 4 specifically contains the scenario of a person who has fainted lying on the floor.

As depicted in Figure 1, two different types of safety clothing were used throughout the experiment. The two garments represent respectively safety class 2 and 3 of the ANSI/ISEA 107-2004 standard and are distinguished by the amount and spatial distribution of the reflective strips.

A synchronized data stream from the NIR stereo camera was recorded at 50 fps, leading to flash/non-flash image pairs available at 25 Hz. In addition, images from a color camera were recorded for the purpose of visualization and to facilitate the manual annotation of the datasets. Several training material datasets are collected in both environments. Both a binary Random Forest and a Support Vector Machine blob classifier are trained on 200 k samples from reflective vest reflectors (positives) and 100 k other reflective objects (negatives). In addition to the camera system, we further equip the sensor unit with a Velodyne HDL-64E 3D LIDAR in order to extract ground truth positions of the tracked persons. All reflectors in the datasets are assigned a manual label whether they represent a safety garment or not. Furthermore, a second label with the ground-truth distance and 3D position of every reflector relative to the camera system is extracted from the LIDAR data. The values of the various system parameters as used throughout the experimental evaluation are listed in Table 1.

### 4.1. Reflector Classification

**Figure 11.**
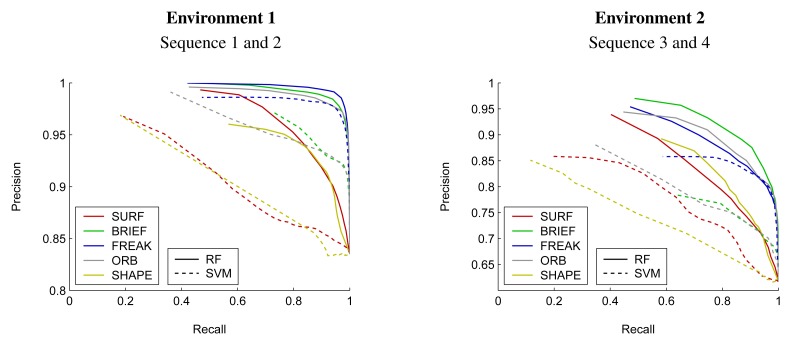
Precision-recall curves showing the classification of individual blobs into reflectors of a safety garment and other reflectors. The curves indicate classification performance as obtained using the state-of-the-art image feature descriptors SURF, BRIEF, FREAK, and ORB in combination with two popular classifier models, a Random Forest (RF) and a Support Vector Machine (SVM). Solid lines represent the output of a Random Forest classifier while the dashed line indicates classification results obtained using a Support Vector Machine.

We first assess the system's ability to discriminate between the reflectors of a safety garment and other reflective objects in the scene. More specifically, we evaluate the supervised learning-based binary classification process as described in Section 3.5, using different choices of state-of-the art image feature descriptors in combination with two popular classifier models, namely a Random Forest and a Support Vector Machine. Figure 11 depicts classification performance in the form of precision-recall curves. The results are regrouped according to the environment in which the data was recorded.

### 4.2. Tracking

We evaluate the output of the tracking module by using criteria defined in the 2D image space. We first manually annotate the trajectories of all humans in every 5th frame of a video sequence. Therefore, we draw bounding boxes around every person's torso and assign a trajectory label to every box. A new trajectory is counted every time a person is occluded during more than 10 consecutive frames. In a single frame, we define a trajectory as *hit* if its bounding box overlaps with the bounding box of a tracked person by more than 50 percent. In the opposite case, the trajectory is defined as *missed* in the given frame. A trajectory that is hit in more than 75% of the frames in which the corresponding person appears is declared *mostly hit*. Similarly, we declare a trajectory as *mostly missed* if it is hit in less than 25% of frames. *The average trajectory coverage* indicates the mean hit ratio over all different trajectories. Finally, we consider *false alarms* that we define as tracked reflective objects that are mistakenly classified as humans. If the same misclassified object appears over several successive frames, we count only one false alarm.

**Figure 12.**
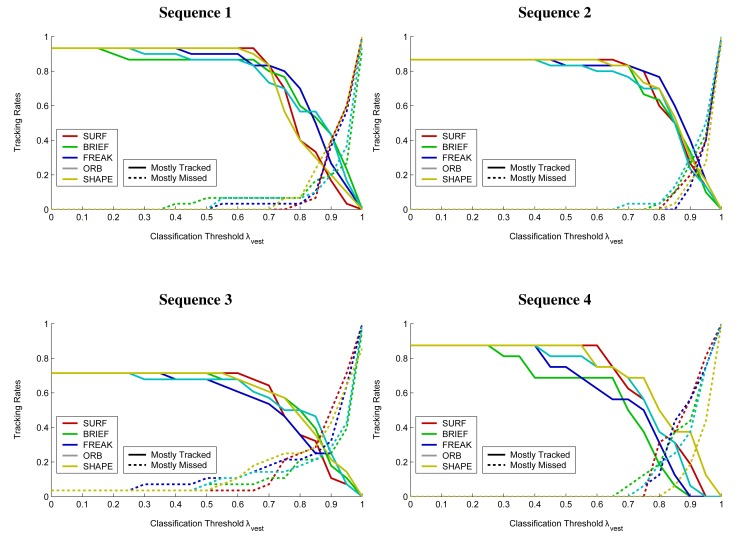
The figures show the tracking rates, defined as the ratios of mostly hit and mostly missed trajectories of humans appearing in the image sequence. The results are shown for different choices of feature descriptors and a varying classification threshold *λ_vest_*.

**Figure 13.**
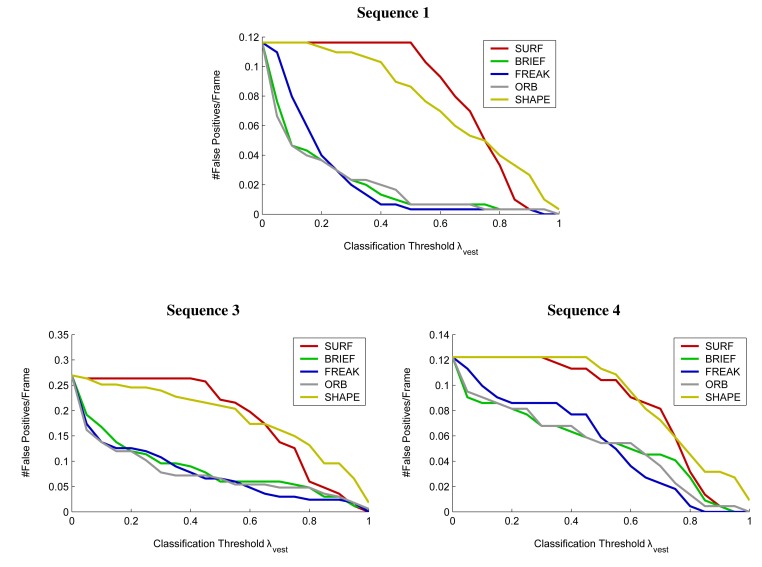
Number of false alarms per frame depending on the feature descriptor and the classification threshold *λ_vest_* in use. No graph is shown for sequence 2, as it does not contain any reflectors other than the ones on the safety garments, leading to zero false alarms independent of the classification threshold.

**Figure 14.**
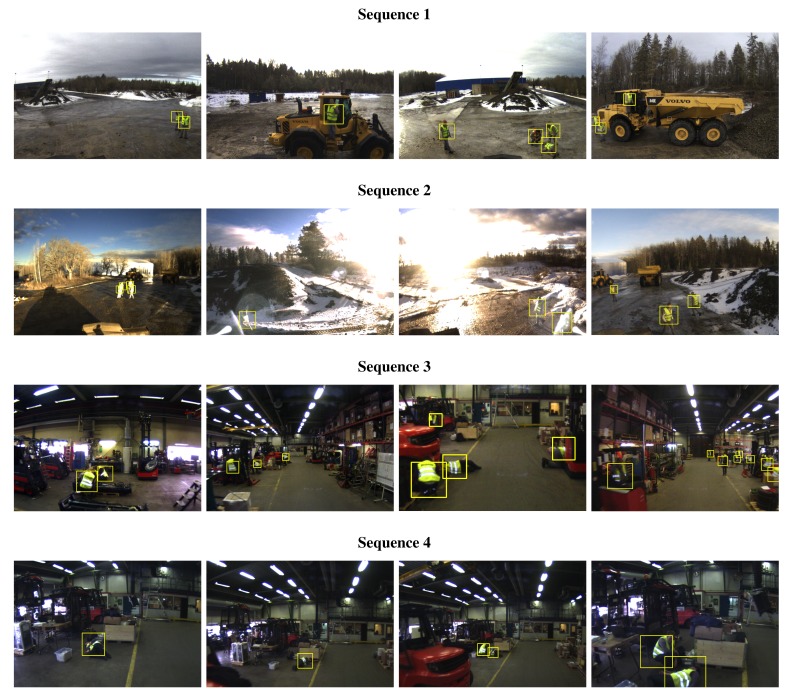
Exemplary tracking results for the test sequences 1–4. The detections obtained from the NIR camera are projected on the color image and indicated with a yellow square.

**Figure 15.**
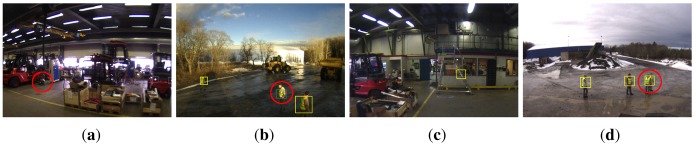
Erroneous tracker outputs: (**a**) missed detection of a person (marked with a red circle) in a body position that hides all reflectors of the safety clothing; (**b**) missed detection of a person that is tracked but misclassified as a non-human; (**c**) false alarms; and (**d**) occasional grouping of persons that stand close to each other.

Tracking performance according to the above measures is shown in Figures 12 and 13 while numerical results for the case of a BRIEF descriptor and a classification threshold of *λ*_vest_ = 0.7 are summarized in Table 2. Only the Random Forest classifier was used for blob classification. Example tracking results are depicted in Figure 14 whereas Figure 15 illustrates different types of erroneous tracker outputs.

### 5. Discussion

Section 4.1 presents an evaluation of the system performance in classifying individual reflective blobs into a group of reflectors originating from safety clothing and a group of other reflectors. The results clearly indicate that a Random Forest is to be preferred to a Support Vector Machine for the underlying classification problem. In terms of the feature descriptor in use, the optimal choice is less clear. brief, freak, and orb descriptors all show good performance. In fact, all three descriptors are of similar nature and consist of binary variables that represent the output of pairwise intensity comparisons at different sampling locations within the interest region. Their performance is similar in both test environments with brief offering the best overall performance. SURF descriptors work less well, even though this is difficult to generalize, as SURF used to rank better in earlier experiments [3].

The tracking results presented in Figures 12, 13 and 14 and Table 2 indicate good performance over all four test scenarios. The evaluated sequences feature multiple persons that often appear in rather unusual, non-upright body positions and were still reliably detected and tracked by the system, even though the classifier was not specifically trained on all possible body poses. The choice of the classification threshold *λ_vest_* is a trade-off between achieving good tracking rates (*cf.* Figure 12) and a low number of false alarms (*cf.* Figure 13). With the exact choice of the value *λ_vest_*, one can give a preference to either the first or the second goal.

Figure 12 also indicates that even for low classification thresholds, the ratio of mostly tracked trajectories is not 100%. A closer analysis of these cases revealed three main reasons. First, as our tracker needs to consistently observe an object over a series of frames before considering it as valid, the coverage of very short trajectories can be significantly reduced. Second, some persons appeared outside the sensor range where no foreground regions could be segmented because the reflected portion of the IR flash was too weak to produce strong enough high-intensity regions. Third, some trajectories represent humans that were occluded to such a high degree that the reflectors on the garment were not or only barely visible.

Our method of tracking industrial workers by observing the reflective patterns on their safety clothing has a number of key advantages over conventional vision-based human detectors. When working in the visible-light range, the performance of detection modules usually decreases gradually as the illumination becomes weaker and the images lose contrast. Especially night time pedestrian detection, therefore, remains an open issue that has been addressed only by limited studies on thermal vision [6]. At the same time, detection can fail in extreme illumination conditions where the camera directly faces a strong light source such as the sun, leading to overexposed images. When evaluating human detection systems, a performance assessment in such exceptional situations is most often left out by the authors. However, for safety critical industrial applications, robust performance also in the most challenging conditions is crucial. During the experimental evaluation, we found our method to cope well with a wide range of different illumination conditions without showing significant performance differences. Especially the results obtained for test sequence 2 illustrate that the system still performs accurately even if the camera setup directly faces the sun. In addition, as we actively illuminate the scene when acquiring the images, our system performs equally well in complete darkness as in broad daylight.

Another strength of our approach is the robustness towards partial occlusion. Existing approaches that are most successful in detecting partially occluded persons are based on models that divide the human body into different parts that are individually detected and classified. On the other hand, we found our own approach to be robust to partial occlusions, even though it is not relying on any complex body appearance model. Several successful detections of partly occluded persons are shown in Figure 14.

As a consequence of the adopted detection method, our system also comes with a certain number of limitations. While the detection of the reflective markers on the worker's safety garments is reliable as long as they are visible to the camera, the method is obviously doomed to fail if the reflectors are fully occluded. In practice, this scenario has only been encountered in very rare occasions though, namely if the target person appears in unusual body positions in which the biggest parts of the safety garment are occluded to the camera (*cf.* Figure 15a). Here, the type of safety clothing in use can play an important role, as garments with a higher safety class are covered with larger areas of reflective material, making a complete occlusion unlikely.

In contrast to other approaches that are able to identify the full silhouette of a person, such as [34], our method also has to deal with the limitation to detect only body parts that are covered with retro-reflective markers. Typically, this is just the case for the upper body while the legs are not detected unless people wear additional reflective safety trousers. By specifically tracking the reflectors of safety clothing, our system further provides alarms regardless of whether or not a garment is actually worn by a person.

By solely observing the reflective patterns of safety garments, it is further difficult to preserve the exact number of tracked persons as well as their identity in cases where two or more humans interact with each other. This is due to the fact that the appearance of the different persons is very similar, especially when working with images that only show the reflective pattern of the safety garments. Here, the use of a conventional visible-light camera could help to better discriminate individual persons from each other.

## 6. Conclusions

In this article, we presented a novel approach for multi-person tracking in industrial work environments in which humans wear reflective safety garments. To the best of our knowledge, our approach is unique in the sense that we perform human tracking solely by observing the reflective patterns that the safety clothing produces in the images acquired with our customized camera setup. Using a monochrome stereo camera setup equipped with flash that operates in the NIR range, we acquire images that considerably simplify the extraction of interest regions in which reflective material appears. Depth and 3D location for the detected reflectors is inferred by locally computing disparities in the area close to the blob contour. Using supervised-learning based classification of the observed reflective patterns, we further discriminate humans wearing a safety garment from other objects with high reflectivity commonly encountered in an industrial work site. The detected reflectors are then regrouped and assigned to objects whose position and speed relative to the camera are recursively estimated over time using particle filtering.

We evaluated our system on two different moving vehicles in challenging industrial indoor and outdoor environments. The results indicate that our approach achieves accurate detection and tracking performance under various and difficult illumination conditions for distances ranging up to 20 m. The robustness towards different illumination conditions offers a clear advantage over many existing human tracking systems. In fact, our camera system operates without significant performance difference in conditions ranging from broad daylight to complete darkness. Furthermore, it has been shown that our system is capable to detect humans not only in upright but in arbitrary body positions. Especially in industrial work sites, this is an important property as a large variety of human body postures are typically encountered.

## Figures and Tables

**Table 1.** Values of the system parameters used throughout the experimental evaluation.

**Image Acquisition and Panoramic Unwrapping**		

Image Pair Acquisition Rate	$f_{a}=T_{a}^{-1}$	25Hz
*I_f_*/*I_nf_* Acquisition Delay	*t_a_*	10ms
Panoramic Image Resolution	*W* × *H*	800 × 530 px
Panoramic Field-of-view	*α*, *β*	90°, 55°

**Segmentation**		

Adaptive Thresholding Window Size	*w_th_* × *w_th_*	21 × 21 px
Adaptive Thresholding Offset	*C_th_*	40
Reflectivity Threshold	*λ*_Δ_	30

**Localization**		

Disparity Extraction Zone	*s*	4

**Tracking**		

Object Consistency Threshold	*λ_μ_*	3
Blob-Object Assignment Cost Function Weights	*W*_∩_, *W*_δ_	1.5, 1.0
Blob-Object Assignment Cost Threshold	*λ_d_*	0.5
Object Classification Threshold	*λ_vest_*	0.7
Measurement Model Uncertainty	*σ_rad_*, *σ_tg_*	0.25, 0.075

**Table 2.** Quantitative tracking results obtained using a BRIEF feature descriptor in combination with a classification threshold. *λ*_vest_ = 0.7

**Sequence**	**Number of Trajectories**	**Mostly Hit Trajectories**	**Mostly Missed Trajectories**	**Average Trajectory Coverage**	**False Alarms**
**1**	30	26	2	80.1%	2
**2**	30	25	0	81.8%	3
**3**	28	18	2	72.2%	10
**4**	16	11	0	77.5%	3
